# 

**Published:** 1913-12

**Authors:** 


J. M. FORTESCUE-BRICKDALE.

### The Library of the Bristol Medico-Chirurgical Society.

*The following donations have been received since the publication of the List in September.*

*November 29th,* 1913.

| | |
|---|---|
| Barclay J. Baron, M.B. (1) .. .. .. | 33 volumes. |
| F. R. Cross, M.B. (2) .. .. .. .. | 5 ,, |
| F. H. Edgeworth, M.D. (3) .. .. .. | 4 ,, |
| L. M. Griffiths (4) .. .. .. .. .. | 25 ,, |
| Victor Horsley (5) .. .. .. .. | 1 volume. |
| The Middlesex Hospital (6) .. .. .. | 1 ,, |
| Sir William Osler (7) .. .. .. .. | 2 volumes. |
| Arthur P. Prowse, M.D. (8) .. .. .. | 1 volume. |
| R. Shingleton Smith, M.D. (9) .. .. .. | 4 volumes. |
| James Swain, M.D. (10) .. .. .. .. | 1 volume. |
| E. T. Wilson, M.B. (11) .. .. .. .. | 67 volumes. |

Pamphlets have been received from Dr. James Swain and Dr. E. T. Wilson.

### NINETIETH LIST OF BOOKS.

The titles of books mentioned in previous lists are not repeated.

The figures in brackets refer to the figures after the names of the donors, and show by whom the volumes were presented. The books to which no such figures are attached have either been bought from the Library Fund, or received through the *Journal.*

**Acland, Sir H. W.** *Oxford and Modern Medicine* .. .. .. (11) 1890
**Allbutt, T. C.** .. *The Harveian Oration* .. .. .. .. (11) 1900
**Andrew, J.** .. .. *The Harveian Oration* .. .. .. .. .. .. 1890
**Andrewes, F. W.**.. *The Croonian Lectures* .. .. .. .. (11) 1910
**Anstie, F. E.** .. *Stimulants and Narcotics* .. .. .. .. 1864
**[Beale, L. S.]** .. *Vitality* .. .. .. .. .. .. .. .. (11) 1899
„ .. .. *The Structure and Formation of Certain Nervous Centres* .. .. .. .. .. (11) 1864
**Benger, [—]** .. .. *Alimentary Enzymes* .. .. .. .. .. (9) 1912
**Bloxam, C. L.** .. *Chemistry* .. .. .. .. .. .. (1) 3rd Ed. 1875
**Bourne, A. W.** .. *Synopsis of Midwifery* .. .. .. .. .. 1913
**Bridges, J. H.** .. *The Harveian Oration* .. .. .. .. (11) 1892
**Bruce, O.** .. .. *Lectures on Tuberculosis for Nurses* .. .. 1913
**Bruce, W.** .. .. *Sciatica* .. .. .. .. .. .. .. .. .. .. 1913
**Burghard, Sir W. W. Cheyne, and F. F.** *A Manual of Surgical Treatment.* (Ed. by T. P. Legg and A. Edmunds) .. .. .. Vol. V, New Ed. 1913
**Carpenter, W. B.**.. *Human Physiology.* (Edited by H. Power.) 8th Ed. 1876
**Carson, T. G.** .. *Man's Responsibility* .. .. .. .. .. .. 1906
**Chambers, T. K.** .. *The Harveian Oration* .. .. .. .. (11) 1871
**Cheyne and F. F. Burghard, Sir W. W.** *A Manual of Surgical Treatment.* (Edited by T. P. Legg and A. Edmunds) .. .. .. Vol. V, New Ed. 1913
**Church, W. S.** .. *The Harveian Oration* .. .. .. .. (11) 1895
**Cleveland, W. F.** *The Modes of Dying* .. .. .. .. .. .. 1870
**Clifford-Smith, J. L.** [Ed.] *Hospital Management* .. .. .. .. .. 1883
**Conn, H. W.** .. *The Story of Germ Life* .. .. .. .. .. .. 1897
**Cornil and Ranvier.** *Pathological Histology* (Trans. by A. M. Hart) .. .. .. .. .. 2 vols (in 3) (1) 1882–86
**Cox, R. [Ed.].** .. *Selections from Phrenological Journal* .. .. 1836
*Deaf, The, Handbook of Information relating to* .. .. .. .. .. 1913
**Degrais, L. Wickham and P.** *Radium and Cancer.* (Trans. by A. and A. G. Bateman) .. .. .. .. .. 1913
**Dickinson, E. H.** *The Medicine of the Ancients* .. .. (11) 1875
**Dickinson, W. H.** *The Harveian Oration* .. .. .. .. (11) 1891
**Dobell, H.** .. .. *Affections of the Heart* .. .. .. .. .. .. 1872
**Donkin, H. B.** .. *The Harveian Oration* .. .. .. .. (11) 1910
**Duckworth, Sir D.** *The Harveian Oration* .. .. .. .. (11) 1898
**Edmunds, W.** .. *Sound and Rhythm* .. .. .. .. .. .. .. 1906
**Eulenberg and P. Guttmann, A.** *Physiology and Pathology of Sympathetic Nervous System.* (Trans. by A. Napier) .. .. .. .. .. .. .. .. .. 1879

| | | |
|---|---|---|
| **Farre, A.** | *The Harveian Oration* .. .. .. .. (11) | 1872 |
| **Ferrier, D.** | *The Harveian Oration* .. .. .. .. .. .. | 1902 |
| **French, H.** | *The Goulstonian Lectures* .. .. .. (11) | 1908 |
| **Galton, D.** | *Construction of Hospitals* .. .. .. .. .. | 1869 |
| **Gillespie, W. R.** | *The Argument, a priori, for the Being and the Attributes of God* .. .. .. .. .. .. | 1906 |
| **Godlee, R. J.** | *Introductory Address* .. .. .. .. .. .. | 1889 |
| **Gordon, W.** | *The Place of Climatology in Medicine* .. .. | 1913 |
| **Gowers, W. R.** | *Epilepsy* .. .. .. .. .. .. .. .. .. .. | 1881 |
| **Greeff, R.** | *Guide to the Microscopic Examination of the Eye.* (Trans. by H. Walker) .. .. .. | [1913] |
| **Green, T. H.** | *Pathology and Morbid Anatomy* (1) 7th Ed. | 1889 |
| **Gull, W. W.** | *The Harveian Oration* .. .. .. .. (11) | 1870 |
| **Guttmann, A. Eulenberg and P.** | *Physiology and Pathology of Sympathetic Nervous System.* (Trans. by A. Napier) .. .. .. .. .. .. .. .. .. | 1879 |
| **Hall, F. de H.** | *Intrathoracic Aneurysm* .. .. .. (11) | 1913 |
| **Harley, G.** | *On Sounding for Gall-stones* .. .. .. .. .. | 1884 |
| **Hamer, W. H.** | *The Milroy Lectures* .. .. .. .. (11) | 1906 |
| **Hardwicke, H. J.** | *Guide to European Universities* .. .. (4) | 1878 |
| **Harris, J. D.** | *Lectures on Medical Electricity to Nurses* .. | 1913 |
| **Harris, T.** | *Post-mortem Handbook* .. .. .. .. .. (1) | 1887 |
| **Harvey, W.** | *Circulation of the Blood.* (Ed. by A. Bowie) | 1889 |
| **Helfft, H.** | *Handbuch der Balneotherapie* 9th Ed. | 1882 |
| **Hénocque, A.** | *Spectroscopie* .. .. .. .. .. .. .. .. (4) | [N.D.] |
| **Hewlett, R. T.** | *The Milroy Lectures* .. .. .. .. .. (11) | 1909 |
| **Hirschfeld and J. B. Leveillé, L.** | *Neurologie* (Texte) .. .. .. .. .. | 1853 |
| **Holland, Sir H.** | *Recollections of Past Life* .. 2nd Ed. | 1872 |
| **Horsley, V.** | *Reprints of Papers by* .. .. .. .. .. (5) | [V.D.] |
| **Hurry, J. B.** | *The Ideals and Organisation of a Medical Society* .. .. .. .. .. .. .. .. .. .. | 1913 |
| **Jamieson, E. B.** | *A Companion to Manuals of Practical Anatomy* | 1913 |
| **Johnson, G.** | *The Harveian Oration* .. .. .. 2nd Ed. | 1883 |
| **Kaltschmidt, J. H.** | *German-English Dictionary* .. .. 2nd Ed. | 1855 |
| **Kenwood, L. C. Parkes, and H. R.** | *Hygiene and Public Health* 5th Ed. | 1913 |
| **Kidd, P.** | *Lumleian Lectures* .. .. .. .. .. .. (11) | 1912 |
| **Landois and W. Stirling, L.** | *Human Physiology.* 2 vols .. (1) | 1885 |
| **Latham, P. W.** | *The Harveian Oration* .. .. .. .. (11) | 1888 |
| **Leveillé, L. Hirschfeld and J. B.** | *Neurologie* (Texte) .. .. .. .. .. | 1853 |
| **Levy, M.** | *Traité d'Hygiène.* 2 vols. .. .. .. (11) | 1862 |
| **Liveing, R.** | *Handbook of Skin Disease* .. .. 5th Ed. | 1887 |
| **Lugaro, C.** | *Modern Problems in Psychiatry.* (Trans. by D. Orr and R. G. Rows) .. [2nd Ed.] | 1913 |
| **Luys, G.** | *Gonorrhœa.* (Trans. and Ed. by A. Foerster) | 1913 |
| **Macdonald, A.** | *Heart Disease and Pregnancy* .. .. .. .. | 1878 |
| **Mackenzie, J.** | *Diseases of the Heart* .. .. .. 3rd Ed. | 1913 |
| **Maclean, C.** | *Epidemic and Pestilential Diseases.* 2 vols | 1817–18 |
| **Miles, A. Thomson and A.** | *Manual of Surgery.* Vol. III 2nd Ed. | 1913 |

**Moxon, S. Wilks and W.** *Pathological Anatomy* .. (1) 3rd Ed. 1889
**Murchison, C.** .. *Continued Fevers* .. .. .. .. .. .. (1) 1862
**Otis, F. N.** .. .. *Clinical Lessons on Syphilis.* 2 Vols. (1) 1883
**Pamphlets.** .. .. 2 Vols. .. .. .. .. .. .. .. .. (11) [V.D.]
**Parkes and H. R. Kenwood, L. C.** *Hygiene and Public Health.* 5th Ed. 1913
**Pollock, J. E.** .. *The Harveian Oration* .. .. .. .. .. 1889
**Poore, G. V.** .. *The Harveian Oration* .. .. .. .. .. 1899
**Pye, [W.].** .. .. *Elementary Bandaging.* (Ed. by W. H. Clayton-Greene and V. Zachary Cope) 13th Ed. 1913
**Pye-Smith, P. H.** .. *The Harveian Oration* .. .. .. .. (11) 1893
**Quekett, J.** .. .. *On the Microscope* .. .. .. (4) 2nd Ed. 1852
**Ramidge, F. H.** .. *Curability of Consumption* .. .. .. .. 1861
**Ranking, W.** .. .. *The Elements of Bandaging* .. .. .. .. 1913
**Ranvier, Cornil and** *Pathological Histology* . (Trans. by A. M. Hart) .. .. .. 2 vols (in 3) (1) 1882–86
**Rigby, E.** .. .. *On Female Diseases* .. .. .. .. .. .. 1857
**Rindfleisch, E.** .. *Lehrbuch der pathologischen Gewebelehre* (1) 5th Ed. 1878
**Roberts, Sir W.** .. *The Harveian Oration* .. .. .. .. (11) 1897
**Schnée, E.** .. .. *Diabetes.* (Trans. by R. L. Tafel) .. .. 1889
**Shepherd, A. B.** .. *Natural History of Pulmonary Consumption* 1877
**Sieveking, E. H.** .. *The Harveian Oration* .. .. .. .. (11) 1877
**Sims, J. M.** .. .. *Uterine Surgery* .. .. .. .. .. .. (4) 1886
*Sketches of Eminent Medical Men* .. .. .. .. .. .. .. .. .. [N.D.]
**Stirling, L. Landois and W.** *Human Physiology.* 2 vols. .. (1) 1885
**Swayne, J. G.** .. *Obstetric Aphorisms.* (Ed. by W. C. Swayne) 11th Ed. 1913
**Tardieu, A.** .. .. *Dictionnaire d'Hygiène publique.* 4 Vols. (11) 2nd Ed. 1862
**Thomson and A. Miles, A.** *Manual of Surgery.* Vol. III. 2nd Ed. 1913
**Thorne, R. T.** .. *Preventive Medicine during the Victoria Era* (11) 1888
**Toogood, J.** .. .. *Reminiscences of Medical Life* .. .. .. 1853
**Verneuil, [—].** .. *De la Gravité des Lesions traumatiques chez les Alcoholiques* .. .. .. .. .. .. (11) 1871
**Virchow, R.** .. .. *Post-mortem Examinations.* (Trans. by T. P. Smith) .. .. .. .. .. .. .. .. (1) 1880
**Walton, H.** .. .. *Diseases of the Eye* .. .. .. .. 3rd Ed. 1875
**Wanklyn, W. McC.** *The Administrative Control of Smallpox* .. 1913
**West, C.** .. .. *Diseases of Women* .. .. .. 4th Ed. 1879
**Wickham and P. Degrais, L.** *Radium and Cancer.* (Trans. by A. and A. G. Bateman) .. .. .. .. .. .. 1913
**Whitla, J. A.** .. *Materia Medica Notes* .. .. .. .. .. 1913
**Wilks and W. Moxon, S.** *Pathological Anatomy* (1) 3rd Ed. 1889
**Wormald, J. and S.** *A Guide to the Mental Deficiency Act* .. .. [N.D.]
**Yeo, I. B.** .. .. *A Manual of Medical Treatment.* (Ed. by R. Crawfurd and E. F. Buzzard.) 2 Vols 5th Ed. 1913
**Younger, D.** .. .. *The Magnetic and Botanic Family Physician* [1887]

### TRANSACTIONS, REPORTS, JOURNALS, &c.

American Association of Obstetricians and Gynecologists, Transactions of the .. .. .. .. .. .. .. .. .. Vol. XXV 1913
American Journal of Obstetrics, The .. .. .. Vol. LXVII 1913
American Journal of the Medical Sciences, The .. Vol. CXLV 1913
American Ophthalmological Society, Transactions of the, Vol. XIII, Pt. 2 1913
Archives of Medicine .. .. .. .. .. .. .. (11) Vols. I–III 1857–62
Archives of the Middlesex Hospital .. .. .. (6) Vol. XXX 1913
Bristol Health Report for 1912 .. .. .. .. .. .. .. .. .. 1913
Bristol Port Sanitary District, Medical Officer's Report for 1912 .. 1913
Department of Neurology, Harvard Medical School, Papers of Vol. V 1912
Edinburgh Medical Journal .. .. .. .. .. .. N.S., Vol. X 1913
Edinburgh Obstetrical Transactions .. .. .. Vol. XXXVIII 1913
Glasgow Medical Journal, The .. .. .. .. Vol. LXXIX 1913
Hospitals—
  Boston City Hospital, Medical and Surgical Reports .. (10) 1913
  Episcopal Hospital Reports, Philadelphia .. .. .. Vol. I 1913
  St. Thomas's Hospital Reports .. .. .. .. Vol. XL 1913
Imperial Cancer Research Fund, Scientific Reports of (2) No. II–V 1905–12
Journal of Experimental Medicine .. .. .. .. Vol. XVII 1913
Journal of Medical Research, The .. .. .. Vol. XXVII 1912–13
Journal of Obstetrics and Gynæcology, The .. .. Vol. XXIII 1913
Local Government Board, Supplement to 35th Annual Report of the (9) 1908
Medical Chronicle, The .. .. .. .. .. .. .. Vol. XXIV 1912–13
Münchener medizinische Wochenschrift .. .. .. .. .. Bd. I 1913
Ophthalmological Transactions .. .. .. .. Vol. XXXIII 1913
Parasitology .. .. .. .. .. .. .. .. .. .. .. .. .. .. .. Vol. V 1912–13
Philippine Journal of Science, The .. .. .. .. Vol. VII 1912
Practitioner, The .. .. .. .. .. .. .. .. .. .. Vol. XC 1913
Progrès médical, Le .. .. .. .. .. .. .. .. .. .. .. .. 1912
Progressive Medicine .. .. .. .. .. .. .. .. .. Vol. III 1913
Public Health .. .. .. .. .. .. .. .. .. .. .. Vol. XXIII 1909–10
Research Laboratory of New York, Studies from .. Vol. VI 1911
Revue hebdomadaire de Laryngologie .. Tom. XXXIV, Vol. I 1913
Royal Academy of Medicine in Ireland, Transactions of the Vol. XXXI 1913
Society of Medical Officers of Health, Transactions of the (11) 1881–84 1886
Surgery, Gynecology and Obstetrics .. .. .. .. .. .. Vol. V 1907

observed during his stay in the hospital. The case illustrated some of the difficulties met with at times in diagnosing abscess of the liver, for the liver had not been enlarged or tender, and the pain had been in the opposite side of the chest. The speaker advocated exploration by abdominal section in such cases, rather than the use of the exploring syringe.

J. Lacy Firth.
A. J. Wright, *Hon. Sec.*

### Local Medical Notes.

### UNIVERSITY OF BRISTOL.

### EXAMINATION RESULTS.

Students of the University have recently passed the following :—

Conjoint Examining Board of London.—*Final Examination.—Medicine :* R. B. Johnson, B. J. L. Fayle. *Surgery :* R. B. Johnson,* B.J. L. Fayle,* C. Kingston,* H. J. D. Smythe.*

L.D.S., R.C.S. Eng.—*Chemistry :* L. B. James. *Physics :* L. B. James, G. M. Haydon, W. G. Milton. *Dental Mechanics :* A. E. Young, C. M. Sheriff, W. A. Culverwell, E. E. Wookey, R. H. Turner. *Dental Metallurgy :* C. M. Sheriff, H. C. R. Aubrey. *Final Examination — Part I :* S. V. Shrimpton. *Part II :* R. H. Basker,* P. W. Flook,* E. W. Thomas.*

F.R.C.S. Eng.—*Primary Examination :* A. G. T. Fisher, D. G. C. Tasker.

University of London.—*M.B., B.S. Examination :* H. J. Orr-Ewing (Gold Medal), C. C. Harrison, E. S. Sowerby, F. W. Clemens. *Group I :* G. M. Campbell. *Group II :* H. J. D. Smythe, A. H. White.

### APPOINTMENTS.

A. Rendle Short, M.D., B.S., B.Sc. Lond., F.R.C.S., Assistant Surgeon to the Bristol Royal Infirmary, has been appointed Hunterian Professor for 1914 and Examiner in Physiology for the Primary Fellowship at the Royal College of Surgeons of England.

* Denotes qualification.

E. W. Hey Groves, M.D., M.S. Lond., F.R.C.S., Surgeon to the Bristol General Hospital, has been appointed Hunterian Professor for 1914 at the Royal College of Surgeons of England.

R. C. Clarke, M.B., Ch.B. Bristol, has been appointed Assistant Physician at the Bristol Royal Infirmary.

H. W. Goodden, M.B., Ch.B. Bristol, M.R.C.S., L.R.C.P., has been appointed Senior Resident Officer at the Bristol Royal Infirmary.

H. J. Orr-Ewing, M.B., B.S. Lond., has been appointed House Physician at the Bristol Royal Infirmary.

E. W. Wade, M.B., B.S. Lond., has been appointed House Surgeon at the Bristol Royal Infirmary.

H. B. Walker, M.B., B.S. Lond., has been appointed House Surgeon at the Bristol Royal Infirmary.

J. C. B. Grant, M.B., Ch.B. Edin., has been appointed Ear, Nose and Throat House Surgeon at the Bristol Royal Infirmary.

N. H. Kettlewell, L.D.S., R.C.S., has been appointed Dental House Surgeon at the Bristol Royal Infirmary.

R. Ll. Jones, M.R.C.S., L.R.C.P., has been appointed House Surgeon at the Bristol General Hospital.

H. W. Parrot, M.B., B.S. Lond., has been appointed House Physician at the Bristol General Hospital.

B. J. L. Fayle, M.R.C.S., L.R.C.P., has been appointed Assistant House Physician at the Bristol General Hospital.

R. B. Johnson, M.R.C.S., L.R.C.P., has been appointed Casualty Officer at the Bristol General Hospital.

R. F. Bolt, M.R.C.S., L.R.C.P., has been appointed Resident Medical Officer at the Queen Victoria Memorial Hospital, Nice, Alpes Maritimes.

C. Kingston, M.R.C.S., L.R.C.P., has been appointed House Surgeon at the Bolingbroke Hospital, Wandsworth Common, S.W.

### LONG FOX LECTURE.

The eighth Long Fox Lecture was delivered at the University on Tuesday, November 18th, by F. H. Edgeworth, M.D., D.Sc., Professor of Medicine. The subject of the Lecture was, "The cranial nerve, their motor nuclei, and muscle sensory fibres." The Vice-Chancellor of the University (Sir Isambard Owen) took the chair. The Lecture will be published in the *Quarterly Journal of Microscopical Science.*

### INDEX.

Accident ? What is an—C. Corfield, 148.

Almond, Dr. G. H.-H.—Urinary products of intestinal intoxication, 130.

Ambulance, The 3rd South Midland Field—Lt.-Col. A. W. Prichard, 159.

Anæmia in older persons, On the course of pernicious—Dr. J. M. Clarke, 97.

Anæsthesia, Intratracheal, new apparatus for, 341, 344, 347.

Anti-meningococcal serum in the treatment of epidemic cerebro-spinal fever, 170.

Arrowsmith, J. W., Obituary notice of, 94.

Ataxia, Three cases of—Dr. J. M. Fortescue-Brickdale, 235.

Bath, Medicine and its practitioners during the earlier years of the history of—Dr. J. Wigmore, 193.

Banti's disease, Splenectomy in—E. W. H. Groves, 331, 382.

Bismuth meals in noting action of purgatives, 353.

Blood-pressures, Abnormally high, 256.

Blood-pressure, Suprarenals and, 48.

**Books, Reviews of**—

Aarons, Dr. S. J.—Gynæcological therapeutics, 179.

Ach, Dr. A.—Beiträge zur ösophagus-chirurgie, 366.

Acromegaly—Dr. L. Mark, 74.

Adami and J. McCrae, Drs. J. G.—A text-book of pathology, 176.

Allen, Dr. R. W.—The bacterial diseases of respiration, 274.

Ambulance, Historical outline of—C. H. Miles, 82.

American Association of Obstetricians and Gynecologists, Transactions of the, 179.

Armstrong, and Dr. J. M. Fortescue-Brickdale, H. G.—A manual of infectious diseases occurring in schools, 278.

Arsenverbindungen und ihre chemotherapeutische bedeutung, Organische—Dr. M. Nierenstein, 71.

Association internationale d'Urologie, Congrès, de l', 84.

Bacteria—Dr. M. Schottelius, 177.

Bacteriology and hæmatology, Clinical—Dr. W. D. Emery, 79.

Ballance, C. A.—Cerebral decompression in ordinary practice, 86.

**Books, Reviews of** (*continued*)—

Bandages, Les—Dr. C. Julliard, 274.

Batten, and H. Thursfield, Drs. A. E. Garrod, F. E. [Eds.].—Diseases of children, 272.

Beatson, Sir G. T.—Modern wound treatment, 370.

Begg, Dr. C.—Sprue: its diagnosis and treatment, 75.

Berry and T. P. Legg, J.—Hare-lip and cleft palate, 59.

Borradaile, L. A.—Manual of elementary zoology, 64.

Brain, Surgery of the skull and—L. B. Rawling, 59.

Bristol Eye Dispensary: a retrospect, 83.

British journal of surgery, 268.

Bruce and W. J. Dilling, Drs. J. M.—Materia medica and therapeutics, 86.

Burghard, Sir W. W. Cheyne and F. F.—A manual of surgical treatment, 66, 365.

Burnet, D. E. —Microbes and toxins, 176.

Bury, Dr. J. S.—Clinical medicine, 88; Diseases of the nervous system, 61.

Cæsarean section in Great Britain and Ireland—Dr. A. Routh, 174.

Cammidge, Dr. P. J.—Glycosuria and allied conditions, 172.

Cancer, The cause of—Dr. J. J. Clarke, 87.

Carling, W. Turner and E. R.—Treatment after operation, 175.

Carter, Dr. A. H.—Elements of practical medicine, 73.

Cerebral decompression in ordinary practice—C. A. Ballance, 86.

Chaplin, Dr. A.—The illness and death of Napoleon Bonaparte, 87.

Cheyne and F. F. Burghard, Sir W. W.—A manual of surgical treatment, 66, 365.

Child, The sexual life of the—Dr. A. Moll, 70.

Childhood, Common disorders and diseases of—Dr. G. F. Still, 85.

Children, Diseases of—Drs. A. E. Garrod, F. E. Batten and H. Thursfield [Eds.], 272.

Children, The medical diseases of—Dr. T. R. C. Whipham, 277.

Chirurgischen untersuchungs-methoden, Die—Dr. H. Gebele, 77.

Choyce, C.C.[Ed.]—A system of surgery, 65.

Clarke, Dr. J. J.—The cause of cancer, 87.

Cleft palate, Hare-lip and—J. Berry and T. P. Legg, 59.

Collie, Dr. A.—Small-pox and its diffusion, 75.

Consumption in general practice—Dr. H. H. Thomson, 57.

Crookshank, Dr. F. G.—Flatulence and shock, 175.

Denslow, Dr. L. N.—The surgical treatment of locomotor ataxia, 85.

Diabetes: its pathological physiology—Dr. J. J. R. MacLeod, 269.

Diagnosis, An index of differential—Dr. H. French [Ed.], 62.

Digestive glands, The work of the—J. P. Pavlov, 61.

**Books, Reviews of** (*continued*)—
Dilling, Drs. J. M. Bruce and W. J.—Materia medica and therapeutics, 86.
Doctor and his work, The—Dr. C. J. Whitby, 56.
Dreams, The interpretation of—Dr. S. Freud, 369.
Emery, Dr. W. D.—Clinical bacteriology and hæmatology, 79.
Encyclopædia of medicine and surgery, The practitioner's—Dr. J. K. Murphy [Ed.], 275.
Eyes, Diseases of the—C. D. Marshall, 368.
Flatulence and shock—Dr. F. G. Crookshank, 175.
Food, The essentials of—Dr. D. Stewart, 81.
Forbes, Dr. J. G.—The pollution of swimming baths, 87.
Fortescue-Brickdale, H. G. Armstrong and Dr. J. M.—A manual of infectious diseases occurring in schools, 278.
French, Dr. H. [Ed.]—An index of differential diagnosis, 62.
Freud, Dr. S.—The interpretation of dreams, 369.
Gabbett, P. C.—Manual for women's voluntary aid detachments, 84.
Gairdner, Life of Sir W. T.—Dr. G. A. Gibson, 63.
Gall-bladder and bile-ducts, Diseases of the liver—Dr. H. D. Rolleston, 276.
Garrod, F. E. Batten and H. Thursfield, Drs. A. E. [Eds.]—Diseases of children, 272.
Gastroscopy, On—W. Hill, 80.
Gebele, Dr. H.—Die chirurgischen untersuchungs-methoden, 77.
Geddes, Dr. G.—Statistics of puerperal fever, 79.
Gibbs, J. H.—Extraction of teeth, 82.
Gibson, Dr. G. A.—Life of Sir W. T. Gairdner, 63.
Glycosuria and allied conditions—Dr. P. J. Cammidge, 172.
Gonococcal infections—C. E. Pollock and L. W. Harrison, 175.
Guy's Hospital reports, 84.
Gynæcological therapeutics—Dr. S. J. Aarons, 179.
Hanna, Dr. W.—Studies in small-pox and vaccination, 279.
Hare-lip and cleft palate—J. Berry and T. P. Legg, 59.
Harrison, C. E. Pollock and L. W.—Gonococcal infections, 175.
Herringham, Dr. W. P.—Kidney diseases, 56.
Hill, W.—On gastroscopy, 80.
Hollander, B.—The first signs of insanity, 67.
Hurry, Dr. J. B.—Vicious circles in disease, 75.
Hutt, C. W.—Hygiene for health visitors, 80.
Hygiene for health visitors—C. W. Hutt, 80.
Immunity and sero-diagnosis, Clinical—Dr. A. Wolff-Eisner, 176.
Infant The nutrition of the—Dr. R. Vincent, 278.
Infectious diseases occurring in schools, A manual of—H. G. Armstrong and Dr. J. M. Fortescue-Brickdale, 278.
Insanity, The first signs of—B. Hollander, 67.
Jamieson, Dr. W. A.—The care of the skin in health, 82.
Julliard, Dr. C.—Les bandages, 274.
Kidney diseases—Dr. W. P. Herringham, 56.
Kriegschirurgie, Leitfaden der praktischen—Dr. W. v. Oettingen, 77.

**Books, Reviews of** (*continued*)—
Laird, J.—Notes on the treatment of tuberculosis, 276.
Lea, Dr. A. W.—Puerperal infection, 178.
Legg, J. Berry and T. P.—Hare-lip and cleft palate, 59.
Lewers, Dr. A. H. N.—Diseases of women, 370.
Liver, gall-bladder and bile-ducts, Diseases of the—Dr. H. D. Rolleston, 276.
Locomotor ataxia, Surgical treatment of—Dr. L. N. Denslow, 85.
Lyth, Dr. E. R.—Influence of thermal environment on the circulation and the body-heat, 270.
McCrae, Drs. J. G. Adami and J.—A text-book of pathology, 176.
McDonagh, J. E. R.—Salvarsan in syphilis and allied diseases, 369.
McKail, Dr. D.—Public health chemistry and bacteriology, 60.
McKendrick, Dr. W. Robertson and A.—Public health law, 80.
Mackie, F. P.—Sleeping sickness, 176.
MacLeod, Dr. J. J. R.—Diabetes: its pathological physiology, 269.
Mark, Dr. L.—Acromegaly: a personal experience, 74.
Marshall, C. D.—Diseases of the eyes, 368.
Martindale and Westcott—The extra pharmacopœia, 77.
Materia medica and therapeutics—Drs. J. M. Bruce and W. J. Dilling, 86.
Medicine, Clinical—Dr. J. S. Bury, 88.
Medicine, Elements of practical—Dr. A. H. Carter, 73.
Medicine, Manual of—Dr. A. S. Woodwark, 88.
Microbes and toxins—Dr. E. Burnet, 176.
Miles, A. Thomson and A.—Manual of surgery, 76.
Miles, C. H.—An historical outline of ambulance, 82.
Moll, Dr. A.—The sexual life of the child, 70.
Morland, Drs. C. Riviere and E.—Tuberculin treatment, 275.
Murphy, J. K. [Ed.]—The practitioner's encyclopædia of medicine and surgery, 275.
Murrell, Dr. W.—What to do in cases of poisoning, 86.
Napoleon Bonaparte, The illness and death of—Dr. A. Chaplin, 87.
Nervous system, Diseases of the—Dr. J. S. Bury, 61.
Neurasthenia, Treatment of, by the teaching of brain control—Dr. R. Vittoz, 78.
Nierenstein, Dr. M.—Organische arsenverbindungen und ihre chemotherapeutische bedeutung, 71.
Nierentuberkulose—Dr. F. Schlagintweit, 271.
Oesophagus-chirurgie, Beiträge zur—Dr. A. Ach, 366.
Oettingen, Dr. W. v.—Leitfaden der praktischen kriegschirurgie, 77.
Operating theatre in private practice, An—C. H. Whiteford, 83.
Operation, Treatment after—W. Turner and E. R. Carling, 175.
Pathology, Text-book of.—Dr. J. G. Adami and J. McCrae, 176.
Pathology, General—M. S. Pembrey and J. Ritchie [Eds.], 367.
Pavlov, J. P.—The work of the digestive glands, 61.
Pembrey and J. Ritchie, M. S. [Eds.]—Text-book of general pathology, 367.
Pharmacopœia, The extra—Martindale and Westcott, 77.

**Books, Reviews of** (*continued*)—
Photographic record and diary, The Wellcome, 87.
Physiology, The new, in surgical and general practice—Dr. A. R. Short, 64.
Pickerill, H. P.—Stomatology, 81.
Poisoning, What to do in cases of—Dr. W. Murrell, 86.
Pollock and L. W. Harrison, C. E.—Gonococcal infections, 175.
Porter, Dr. W. G.—Diseases of the throat, nose and ear, 368.
Prichard, E. A.—The Bristol Eye Dispensary, 1812-1912, 83
Public health chemistry and bacteriology —Dr. D. McKail, 60.
Public health law—Dr. W. Robertson and A. McKendrick, 80.
Puerperal fever, Statistics of—Dr. G. Geddes, 79.
Puerperal infection—Dr. A. W. Lea, 178.
Pye's surgical handicraft, 89.
Rawling, L. B.—Landmarks and surface markings of the human body, 79; The surgery of the skull and brain, 59.
Rectum, Surgery of the—Sir F. Wallis, 67.
Respiration, The bacterial diseases of, and vaccines in their treatment—Dr. R. W. Allen, 274.
Rice, Dr. A. G.—Medical inspection of schools, 277.
Ritchie, M. S. Pembrey and J. [Eds.]—Text-book of general pathology, 367.
Riviere and E. Morland, Drs. C.—Tuberculin treatment, 275.
Robertson and A. McKendrick, Dr. W—Public health law, 80.
Rolleston, Dr. H. D.—Diseases of the liver, gall-bladder and bile-ducts, 276.
Routh, Dr. A.—Cæsarean section in Great Britain and Ireland, 174.
St. Bartholomew's Hospital re[illegible] 83.
Salvarsan in syphilis and allied diseases—J. E. R. McDonagh, 369.
Schlagintweit, Dr. F.—Technik der diagnose, operation und harnleiterbehandlung bei nierentuberkulose, 271.
Schools, Infectious diseases occurring in—H. G. Armstrong and Dr. J. M. Fortescue-Brickdale, 278.
Schools, Medical inspection of—Dr. A. G. Rice, 277.
Schottelius, Dr. M.—Bacteria, 177.
Sero-diagnosis, Clinical immunity and—Dr. A. Wolff-Eisner, 176.
Sexual life of the child, The—Dr. A. Moll, 70.
Shock, Flatulence and—Dr. F. G. Crookshank, 175.
Short, Dr. A. R.—The new physiology in surgical and general practice, 64.
Skin, Care of, in health—Dr. W. A. Jamieson, 82.
Skull and brain, Surgery of the—L. B. Rawling, 59.
Sleeping sickness—F. P. Mackie, 176.
Small-pox and its diffusion—Dr. A. Collie, 75.
Small-pox and vaccination, Studies in—Dr. W. Hanna, 279.
Small-pox, How to diagnose — W. McC. Wanklyn, 278.
Sprue, its diagnosis and treatment—Dr. C. Begg, 75.
Stewart, Dr. D.—The essentials of food, 81.
Still, Dr. G. F.—Common disorders and diseases of childhood, 85.
Stomatology—H. P. Pickerill, 81.
Surface markings of the human body, Landmarks and—Dr. L. B. Rawling, 79.

**Books, Reviews of** (*continued*)—
Surgery, British journal of, 268.
Surgery, Manual of—A. Thomson and A. Miles, 76.
Surgery, System of—C. C. Choyce [Ed.], 65.
Surgical handicraft, Pye's, 89.
Surgical treatment, Manual of—Sir W. W. Cheyne and F. F. Burghard, 66, 365.
Swimming baths, The polution of—Dr. J. G. Forbes, 87.
Teeth, Extraction of—J. H. Gibbs, 82.
Thermal environment on the circulation and body heat, Influence of —Dr. E. R. Lyth, 270.
Thomson and A. Miles, A.—Manual of surgery, 76.
Thomson, H. H. —Consumption in general practice, 57.
Throat, nose and ear, Diseases of the—Dr. W. G. Porter, 368.
Thursfield, Drs. A. E. Garrod, F. E. Batten, H. [Eds.]—Diseases of children, 272.
Treatment after operation—W. Turner and E. R. Carling, 175.
Tuberculin treatment—Drs. C. Riviere and E. Morland, 275.
Tuberculosis, Notes on the treatment of—J. Laird, 276.
Turner and E. R. Carling, W.—Treatment after operation, 175.
Urologie, Congrès de l'Association internationale d', 84.
Vaccination, Studies in small-pox and—Dr. W. Hanna, 279.
Vaccines in the treatment of the bacterial diseases of respiration—Dr. R. W. Allen 274.
Vicious circles in disease—Dr. J. B. Hurry, 75.
Vincent, Dr. R.—The nutrition of the infant, 278.
Vittoz, Dr. R.—Treatment of neurasthenia by the teaching of brain control, 78.
Wallis, Sir F.—Surgery of the rectum, 67.
Wanklyn, W. Mc C.—How to diagnose small-pox, 278.
Westcott, Martindale and—The extra pharmacopœia, 77.
Whipham, Dr. T. R. C.—The medical diseases of children, 277.
Whitby, Dr. C. J.—The doctor and his work, 56.
Whiteford, C. H.—An operating theatre in private practice, 83.
Wolff-Eisner, Dr. A.—Clinical immunity and sero-diagnosis, 176.
Women, Diseases of—Dr. A. H. N. Lewers, 370.
Women's voluntary aid detachments, Manual for—P. C. Gabbett, 84.
Woodwark, Dr. A. S.—Manual of medicine, 88.
Wound treatment, Modern—Sir G. T. Beatson, 370.
Zoology, A manual of elementary—L. A. Borradaile, 64.

Brachial hypertrophy, 189.
Brasher, Dr. C. W. J.—A case of alternating oxaluria and phosphaturia, 21; Intermittent swelling of the parotid gland, 94, 238.
Bristol Medico-Chirurgical Society, 93, 186, 380.

Calculus, Two cases of urinary—C. A. Moore, 126.
Cancer, Methods of diagnosis in gastric—Dr. J. M. Fortescue-Brickdale, 108.
Cardiac lesion, Case of congenital, 188.
Carwardine, T.—Pericolitis, 333.
Cerebral symptoms of lobar pneumonia in children—Dr. F. H. Edgeworth, 308.
Cerebro-spinal fever, Anti-meningococcal serum in the treatment of epidemic, 170.
Cerebro-spinal meningitis, Some recent cases of epidemic—Dr. J. R. Charles, 142.
Cervical rib, Case of—Dr. R. Waterhouse, 232.
Cervical ribs, 165.
Charles, Dr. J. R.—Some recent cases of epidemic cerebro-spinal meningitis, 142.
Children, The cerebral symptoms of lobar pneumonia in—Dr. F. H. Edgeworth, 308.
Clarke, Dr. J. M.—Case of acute septicæmia due to B. pyocyaneus, 4; On the course of pernicious anæmia in older persons, 97; Report on medicine, 48.
Clifton Zoological Gardens, Medical observations in—Dr. A. J. Harrison, 318.
Constipation, Discussion on the treatment of, 44.
Constipation, The treatment of chronic—Dr. F. H. Edgeworth, 40.
Coombs, Dr. C.—An example of Stokes-Adams syndrome, 30.
Corfield, C.—What is an accident? 148.

Diagnosis in gastric cancer, Methods of—Dr. J. M. Fortescue-Brickdale, 108.

Edgeworth, Dr. F. H.—Notes on a case of alternating oxaluria and phosphaturia, 18; On the occurrence of serous pleurisy due to pneumococci and tubercle bacilli, 146; The treatment of chronic constipation, 40; On the cerebral symptoms of lobar pneumonia in children, 308.
Editorial note, 371.
Ergot, Variation in the preparations of, 279.
Ether, Apparatus for intratracheal administration of—Dr. F. E. Shipway, 341.
Eye disease caused by toxæmia, 265.

Ferments, Protective—Dr. I. W. Hall, 304.
Fibrositis, 260.
Field ambulance, 3rd South Midland—Lt.-Col. A. W. Prichard, 159.
Firth, Dr. J. L.—On nephropexy, 220.
Flemming, C. E. S.—Paroxysmal œdema of the lungs, 121.
Fortescue-Brickdale, Dr. J. M.—Methods of diagnosis in gastric cancer, 108; The causes of variations in the preparations of ergot, 279; Three cases of ataxia, 235. Therapeutic notes, 279, 373; Report on medicine, 350.
Fox (Long) lecture, 384.
Fry, Dr. S. V. Stock, and J. D.—A combined manometer and safety valve for intratracheal anæsthesia, 344, 348.

Galactogogues, old and new, 373.
Gallstones, The end-results of cases operated on for—Dr. A. R. Short, 34.
Gastric and duodenal ulceration, 351.
Gastric cancer, Methods of diagnosis in—Dr. J. M. Fortescue-Brickdale, 108.
Gonorrhœa, Recent treatment of, 354.
Groves, E. W. H.—Report on surgery, 53; A simple apparatus for intratracheal anæsthesia, 347; Splenectomy in Banti's disease, 331, 382.

Hall, Dr. I. W.—Mixed infections, 227; Protective ferments, 304.
Harrison, Dr. A. J.—Medical observations in the Clifton Zoological Gardens, 318.
Hemorrhagic diseases of the new-born—Dr. W. J. H. Pinniger, 248.
Hexamethylenamin in treatment of system infections, 49.
Hypernephroma or mesothelioma of the kidney—Dr. J. Swain, 213.

Infections, Mixed—Dr. I. W. Hall, 227.

Intestinal intoxication, Urinary products of—Dr. G. H.-H. Almond, 130.
Intratracheal administration of ether, Apparatus for—Dr. F. E. Shipway, 341.
Intratracheal anæsthesia, A simple apparatus for—E. W. H. Groves, 347.
Intratracheal anæsthesia, Combined manometer and safety valve for—Dr. S. V. Stock and J. D. Fry, 344, 348.

Jaundice, Epidemic catarrhal, 50, 52.
Jenkins, H. S., Obituary notice of, 189.

Kidney, Hypernephroma or mesothelioma of the—Dr. J. Swain, 213.

Leukæmia, Aleucocythæmic—Dr. R. Waterhouse, 10.
Library of the Bristol Medico-Chirurgical Society, 91, 183, 285, 375, 380.
Liver, Case of abscess of the, 382.
Local medical notes, 95, 192, 287.
Luetin reaction, Intradermic, 169.
Lungs, Paroxysmal œdema of the—C. E. S. Flemming, 121.

Medical curriculum, Specialism and the — Dr. P. Watson-Williams, 289.
Medicine, Reports on—Dr. J. M. Clarke, 48 ; Dr. G. Parker, 256 ; Dr. J. M. Fortescue-Brickdale, 350.
Meningitis, Recent cases of epidemic cerebro-spinal—Dr. J. R. Charles, 142.
Migraine, Etiology of, 187.
Moore, C. A.—Notes on two cases of urinary calculus, 126.
Muscular atrophy, Juvenile type of, 188.
Myopia, Causation of, 266.

Neo-salvarsan in syphilis, Intravenous administration of—Dr. J. A. Nixon, 24.
Nephropexy—Dr. J. L. Firth, 220.
New-born, On hemorrhagic diseases of the—Dr. W. J. H. Pinniger, 248.
Nixon, Dr. J. A.—The intravenous administration of neo-salvarsan in syphilis, 24 ; Splenectomy for splenomegaly, 325.
Nurses, The training of, 371.

Obituary, 94, 189, 287.
Œdema of the lungs, Paroxysmal—C. E. S. Flemming, 121.
Older persons, On the course of pernicious anæmia in—Dr. J. M. Clarke, 97.
O'Leary, Rev. de L.—The ethical aspects of physical research, 242.
Ophthalmitis, Sympathetic, 268.
Ophthalmology, Report on—C. H. Walker, 265.
Otology, Report on—Dr. P. Watson-Williams, 360.
Oxaluria and phosphaturia, Cases of alternating—Dr. C. W. J. Brasher, 21 ; Dr. F. H. Edgeworth, 18.

Pancreatic secretion, 350.
Parker, Dr. G.— Report on medicine, 256.
Parotid gland, Intermittent swelling of the—Dr. C. W. J. Brasher, 94, 238.
Pathology, Report on — G. S. Williamson, 168.
Pericardiac sounds, Certain adventitious, 186.
Pericolitis—T. Carwardine, 333.
Phosphaturia, Cases of alternating oxaluria and—Dr. C. W. J. Brasher, 21 ; Dr. F. H. Edgeworth, 18.
Pinniger, Dr. W. J. H.—On hemorrhagic diseases of the new-born, 248.
Pituitary body, Tumours and disease of, 361.
Pleurisy due to pneumococci and tubercle bacilli, On the occurrence of serous—Dr. F. H. Edgeworth 146.
Pneumococci and tubercle bacilli, On the occurrence of serous pleurisy due to—Dr. F. H. Edgeworth, 146.
Pneumonia in children, Cerebral symptoms of—Dr. F. H. Edgeworth, 308.
Poliomyelitis, The epidemiology of epidemic, 171.
Pregnant uterus, Rupture of, before labour, 94.
Prichard, Lt.-Col. A. W.—The 3rd South Midland Field Ambulance, 159.

Purgatives, Action of, 353.
Pyocyaneus, Case of acute septicæmia due to B.—Dr. J. M. Clarke, 4.

Radium emanation, 259.
Research, The ethical aspects of physical—Rev. de L. O'Leary, 242.
Rhinology, Report on—Dr. P. Watson-Williams, 360.
Rhinorrhœa, Cerebro-spinal, 187.

Salpingoscopy, Direct, 360.
Septicæmia due to B. pyocyaneus, Case of—Dr. J. M. Clarke, 4.
Shipway, Dr. F. E.—An apparatus for the intratracheal administration of ether, 341.
Shock, Nature of surgical, 187.
Short, Dr. A. R.—Report on surgery, 262 ; The end-results of cases operated on for gallstones, 34.
Short, Dr. L. J.—The tuberculosis medical officer and his work, 153.
**Sick, Preparations for the—**
Afridol soap, 181.
Bolus soap, 90.
Chrismol, 181.
Ems mineral spring salt pastilles, 182.
Euthymol preparations, 181.
Fortovim, 282.
Glycero-phosphates : rubelix, 89.
Helmitol Co. tablets, 283.
Horlick's malted milk tablets, 180.
Hydrastinin-hydrochl. tablets, 283.
Kalari biscuits, 284.
Lactona, 182.
Luminal, 181.
Luminal sodium, 181.
Novatophan, 182.
Œthone-falcoz, 285.
Phosphocose, 180.
Pond's tampons, 182.
Tabloid thyroid gland, 90.
Tuberculin bouillon filtrate, 90.
Validol and compounds, 283.
Visem, 282.
Wander malt extact, 284.
Winox, 285.
Skin grafting, 54.
Smith, Dr. R. Shingleton, Dinner to, 1.
Society, Bristol Medico-Chirurgical, 93, 186, 380.
Specialism and the medical curriculum—Dr. P. Watson-Williams, 289.
Splenectomy in Banti's disease—E. W. H. Groves, 331, 382.
Splenectomy for splenomegaly—Dr. J. A. Nixon, 325.
Splenomegaly, Splenectomy for—Dr. J. A. Nixon, 325.
Splints, Arm, 187.
Stevens, W. H., Obituary notice of, 287.
Stock and J. D. Fry, Dr. S. V.—A combined manometer and safety valve for intratracheal anæsthesia, 344, 348.
Stokes - Adams syndrome, An example of—Dr. C. Coombs, 30.
Suprarenals and blood-pressure, 48.
Surgery, Reports on—E. W. H. Groves, 53 ; Dr. A. R. Short, 262 ; Dr. J. Swain, 165 ; C. F. Walters, 354.
Swain, Dr. J.—Hypernephroma or mesothelioma of the kidney, 213 ; Report on surgery, 165.

Tetanus, Pathology and treatment of, 262.
Therapeutic notes, 279, 373.
Tissue-cells, Cultivation of, *in vitro*, 172.
Toxæmia as a cause of eye disease, 265.
Transplantation of tissues and organs, 53.
Treponema pallidum, Culture of the, 168.
Tuberculin reactions, 169.
Tuberculosis medical officer and his work, The—Dr. L. J. Short, 153.

Urinary products of intestinal intoxication—Dr. G. H.-H. Almond, 130.
University of Bristol, 96.

Viscera, Grafting of, 55.

Walker, Dr. C. H.—Report on ophthalmology, 265.
Walters, C. F.—Report on surgery, 354.
Waterhouse, Dr. R.— Aleucocythæmic leukæmia, 10 ; A case of cervical rib, 232.
Watson-Williams, Dr. P.—Report on otology and rhinology, 360 ; Specialism and the medical curriculum, with special reference to otology, rhinology and laryngology, 289.
Wigmore, Dr. J.—Medicine and its practitioners during the earlier years of the history of Bath, 193.
Williamson, G. S.—Report on pathology, 168.

X-rays in gastric ulceration, 352.

Zoological Gardens, Medical observations in the—Dr. A. J. Harrison, 318.

### *PUBLICATIONS RECEIVED.*

From Adlard and Son, London :
*Radium and Cancer.* By Louis Wickham, M.V.O., and Paul Degrais. Translated by A. and A. G. Bateman, M.B., C.M. 1913. Price, 2s. 6d. net.

From Baillière, Tindall and Cox, London :
*A Text-Book on Gonorrhea.* By Dr. Georges Luys. Translated and Edited by Arthur Foerster, M.R.C.S. 1913. Price, 15s. net.
*Sciatica.* By William Bruce, M.A., LL.D., M.D. 1913. Price, 5s. net.

From Cassell and Company, Limited, London :
*A Manual of Medical Treatment.* By I. Burney Yeo, M.D. Fifth Edition, by Raymond Crawfurd, M.A., M.D., and E. Farquhar Buzzard, M.A., M.D. Two volumes. 1913. 25s. net.

From J. & A. Churchill, London :
*Obstetric Aphorisms.* By Joseph Griffiths Swayne, M.D. Eleventh Edition. Revised and Edited by Walter Carless Swayne, M.D., B.S. 1913. Price, 3s. 6d. net.
*The Ideals and Organisation of a Medical Society.* By Jamieson B. Hurry, M.A., M.D. 1913. 2s. net.

From W. J. Dornan, Philadelphia :
*Medical and Surgical Reports of the Episcopal Hospital.* Volume I. 1913.

From Henry Frowde & Hodder & Stoughton, London :
*Diseases of the Heart.* By James Mackenzie, M.D., F.R.C.P. Third Edition. 1913. Price, 25s. net.
*A Companion to Manuals of Practical Anatomy.* By E. B. Jamieson, M.D. 1913. Price, 6s. net.
*The Elements of Bandaging and the Treatment of Fractures and Dislocations.* By William Ranking, M.A., M.B., Ch.B. 1913. 5s. net.
*Manual of Surgery.* By Alexis Thomson and Alexander Miles. Second Edition. Vol. III. 1913. Price, 10s. 6d. net.

From P. S. King & Son, London :
*The Deaf: A Handbook containing information relating to Institutions for the Deaf.* 1913. Price, 6d.
*A Guide to the Mental Deficiency Act,* 1913. By John Worwald and Samuel Worwald. [n.d.] 5s. net.

From H. K. Lewis, London :
*The Place of Climatology in Medicine.* By William Gordon, M.A., M.D. 1913. Price, 3s. 6d. net.
*Hygiene and Public Health.* By Louis C. Parkes, M.D., D.P.H., and Henry R. Kenwood, M.B., D.P.H. Fifth Edition. 1913. 12s. 6d. net.
*Lectures on Medical Electricity to Nurses.* By J. Delpratt Harris, M.D. 1913. Price, 3s. 6d. net.
*Lectures on Tuberculosis to Nurses.* By Olliver Bruce. 1913. 2s. 6d. net.

From E. & S. Livingstone, Edinburgh :
*Materia Medica Notes.* By James A. Whitla. 1913. Price, 2s. 6d. net.

From Longmans, Green, and Co., London :
*The Administrative Control of Smallpox.* By W. McC. Wanklyn, B.A., D.P.H. 1913. Price, 3s. 6d. net.
*A Manual of Surgical Treatment.* By Sir W. Watson Cheyne, Bart., C.B., and F. F. Burghard, M.S., F.R.C.S. New Edition. Entirely revised with the assistance of T. P. Legg, M.S., F.R.C.S., and Arthur Edmunds, M.S., F.R.C.S. Volume V. 1913. Price, 21s. net.

From "The Ophthalmoscope" Press, London :
*Guide to the Microscopic Examination of the Eye.* By Professor R. Greeff, with the co-operation of Professor Stock and Professor Wintersteiner. Translated from Third German Edition by Hugh Walker, M.A., M.B. [1913.] Price, 7s. 6d. net.

From The University Press, Manchester :—
*Modern Problems in Psychiatry.* By Ernesto Lugaro. Translated by David Ord, M.D., and R. G. Rows, M.D. [Second Edition.] 1913. Price, 7s. 6d. net.

From John Wright & Sons Ltd., Bristol :
*Pye's Elementary Bandaging and Surgical Dressing.* Revised and partly re-written by W. H. Clayton-Greene, B.A., M.B., B.C. Assisted by V. Zachary Cope, B.A., M.D., M.S. Thirteenth Edition. 1913. Price, 2s. net.
*Synopsis of Midwifery.* By Aleck W. Bourne, B.A., M.B., B.C., F.R.C.S. 1913. Price, 5s. net.

